# Risk factors for treatment failure of surgical site deep infections following spinal instrumentation: multicentre observational study (DeFEndO study)

**DOI:** 10.5194/jbji-11-601-2026

**Published:** 2026-10-06

**Authors:** Renato Pascale, Mena Gallo, Nicolò Regge Gianas, Giacomo Fornaro, Irene Grassi, Manuel Zagarrigo, Andrea Grechi, Alberto Zuppiroli, Dario Santoro, Elena Tenti, Luca Boriani, Eleonora Zamparini, Alessandro Gasbarrini, Pierluigi Viale, Maddalena Giannella, Stefania Curti, Sara Tedeschi

**Affiliations:** 1 Department of Medical and Surgical Sciences, Alma Mater Studiorum University of Bologna, Bologna, Italy; 2 Infectious Diseases Unit, Department for Integrated Infectious Risk Management, IRCCS Azienda Ospedaliero-Universitaria di Bologna, Bologna, Italy; 3 Unit of Neurosurgery, Maria Cecilia Hospital, GVM Care & Research, Cotignola, Ravenna, Italy; 4 Maria Cecilia Hospital, GVM Care and Research, Cotignola, Ravenna, Italy; 5 Spine Surgery, IRCCS Istituto Ortopedico Rizzoli, Bologna, Italy; 6 Department of Biomedical and Neuromotor Sciences, University of Bologna, Bologna, Italy

## Abstract

**Introduction**: To describe the characteristics and outcome of patients diagnosed with deep surgical-site infections following spinal instrumentation (DSIIs) and to investigate risk factors for treatment failure including antimicrobial treatment duration as the main exposure. **Materials and methods**: An observational retrospective study on patients diagnosed with DSII from January 2018 to June 2022 in two large hospitals. Two multivariable analyses were carried out to evaluate risk factors for treatment failure considering two treatment schedules: 8-week versus 12-week. **Results**: We enrolled 161 patients, median age 62 years (IQR: 51–69), 55.1 % male, median Charlson comorbidity index of 3 (IQR: 1–4). Gram-positive and Gram-negative bacteria were responsible for DSII in 60.3 % and 33.8 % of cases. Polymicrobial infections accounted for 42 cases (26.4 %). Treatment failure was observed in 51 (31.7 %) patients. Multivariable analyses were conducted to evaluate risk factors for treatment failure using treatment duration cutoffs of 8 weeks and 12 weeks. Quinolone administration was associated with a lower risk of treatment failure in both treatment schedules (8 weeks: OR 0.11, 95 % CI 0.02–0.71, p=0.020 ; 12 weeks: OR 0.11, 95 % CI 0.02–0.64, p=0.015 ). Gram-negative aetiology of DSII was a risk factor for treatment failure (8 weeks: OR 4.20, 95 % CI 1.08–16.38, p=0.039 ; 12 weeks: OR 4.46, 95 % CI 1.15–17.23, p=0.030 ). **Conclusions**: DSII caused by Gram-negative bacteria was associated with a higher risk of worse outcomes. Quinolone use appeared to be associated with a lower risk of treatment failure, but this finding should be interpreted with caution given the potential for residual confounding. No significant association between treatment duration and outcome was observed in our exploratory analyses.

## 1 Introduction

Deep infections following spinal instrumentation (DSII) are a major complication with mortality rates greater than 10 % and long-term morbidity due to prolonged hospitalization and the need of multiple surgical revisions (Buser et al., 2018; de la Hera et al., 2021; Yao et al., 2018). The optimal management of DSII requires surgical treatment followed by prolonged antibiotic therapy. Surgical treatment can be conservative, with debridement without implant removal, or more aggressive, with complete replacement/removal of the instrumentation, depending on the clinical patient's characteristics and the type of infection (Tai et al., 2024; Zimmerli and Ochsner, 2003). The choice and duration of antimicrobial therapy for DSII is based on microorganism susceptibility as well as the patient's characteristics (de la Hera et al., 2021; Rodríguez-Baño et al., 2018) Usually, a total duration of 8–12 weeks of antibiotic treatment is reported in the literature (de la Hera et al., 2021; Spatenkova et al., 2021; Tai et al., 2024). However, short-term treatments are reported (Benavent et al., 2021; Fernandez-Gerlinger et al., 2019; Karamian et al., 2022) Therefore, the optimal treatment duration is still a matter of debate.

The aims of our study are to describe the characteristics and outcome of patients diagnosed with DSII in two high-volume hospitals and to define risk factors for treatment failure including antimicrobial treatment duration as the main exposure.

## 2 Materials and methods

### 2.1 Study design and setting

This is a retrospective, multicentre study on patients diagnosed with DSII from January 2018 to June 2022, managed at two referral centres for bone and joint infection: the Spine Surgery Unit of the IRCCS Istituto Ortopedico Rizzoli (Bologna, Italy) and the Neurosurgery Unit of Maria Cecilia Hospital, GVM Care & Research (Cotignola, Ravenna, Italy). All patients with DSII underwent surgical debridement followed by antimicrobial therapy. A dedicated ID consultant team was responsible for the DSII diagnosis process and the prescription and monitoring of antibiotic therapy.

Empirical broad-spectrum antibiotic therapy was started immediately after surgery, including coverage of methicillin-resistant *Staphylococcus* spp. plus an agent active against Gram-negative rods; whenever possible, antibiotic therapy was de-escalated according to culture results and continued up to 12 weeks after surgical debridement. Treatment duration was defined by the ID consultant managing the patient and not dictated by study procedures. The choice of surgical strategy (implant retention, removal, or substitution) was individualized according to patient characteristics, primarily based on the assessment of spinal stability (more details in the Supplement).

Data were collected from hospital records and inserted in a dedicated REDCap electronic case report form (eCRF) hosted by IRCCS Azienda Ospedaliero-Universitaria di Bologna (Harris et al., 2019). The study was conducted according to the Declaration of Helsinki and good clinical practice guidelines and was approved by the local ethics committee (no. 712/2022/Oss/AOUBo).

### 2.2 Population

All adult ( ≥18 years) patients diagnosed with DSII and hospitalized at study centres during the study period were screened for inclusion using local registries. Patients' follow-ups were at minimum 360 d after the DSII diagnosis.

### 2.3 Variables and definitions

DSII is defined as infections at surgical site involving all tissues underneath the fascia. DSII diagnosis was established according to a recent Delphi consensus definition (Shaw et al., 2025). Specifically, DSII was defined using a hierarchical set of criteria considering microbiological, clinical, radiological, laboratory, intraoperative, and histological findings. The identification of the same microorganism in at least two deep operative-site specimens was considered diagnostic of infection alone. In its absence, the diagnosis was based on combinations of primary and secondary supporting criteria (Shaw et al., 2025). DSII diagnosis was carried out by a dedicated team of ID specialists (more details in the Supplement). DSIIs were classified as early infections if they appeared in the 3 months after the first surgery, delayed infections between 3 and 24 months, and late infections more than 24 months thereafter (Zimmerli and Ochsner, 2003). Patients were excluded from the study in the case of unavailability of clinical data or follow-up of less than 1 year.

The primary endpoint was treatment failure defined by one or more of the following conditions after appropriate combined medical and surgical treatment: spontaneous reopening of the wound, onset/recurrence of fistula, new onset of pain/clinical impairment developed after a stable resolution following surgical debridement, death related to DSII. Treatment failure was confirmed by ID evaluation to exclude any other cause than relapse of infection due to the same pathogen of the first DSII episode. In cases of suspected mechanical complications, the assessment was integrated with orthopaedic evaluation to ensure appropriate differentiation between infectious and non-infectious causes. Treatment failure was defined as early if detected during antibiotic treatment and late if recorded after the conclusion of antibiotic treatment. Surgical management was classified as removal of spine instrumentation without repositioning, one-stage revision of spine instrumentation (removed and repositioned in a single intervention), or surgical debridement without or with partial replacement (e.g. vertebral cages fixed in place). A full list of definitions and variables is reported in the Supplement.

### 2.4 Statistical analysis

Continuous variables were expressed as median and interquartile range (IQR) and compared using the Mann–Whitney U test. The assumption of normality of the variables was tested through the skewness and kurtosis test for normality as well as visual inspections. Categorical variables were reported as counts and percentages and compared with Pearson's $\chi^{2}$ test or Fisher's exact test, as appropriate. Comparison of patients' characteristics with and without treatment failure was performed. Antibiotic treatment duration was considered as the main exposure variable. According to commonly recommended cutoffs in the literature, (Tai et al., 2024) two binary variables were defined, namely proposed treatment duration (8-week duration) and standard treatment duration (12-week duration). To investigate the effect of a complete antibiotic course, patients were classified into early failure (i.e. diagnosed during antibiotic treatment) and late failure (i.e. recorded after the conclusion of antibiotic treatment). Main multivariable analyses were carried out among those patients with late failure. A set of a priori variables were chosen for the main analysis as risk factors for treatment failure, namely cancer as the reason of surgery, age, Charlson comorbidities index (CCI), presence of polymicrobial infection, quinolone and/or rifampin antibiotic treatment, and etiological agent group. These variables were entered in two separate multivariable logistic regression models according to 8 and 12 weeks of antibiotic treatment duration, respectively. Age and CCI were entered as binary variables on the basis of Youden's cutoffs (Fluss et al., 2005). A complete case analysis was performed. Odds ratios (ORs) and their corresponding 95 % confidence intervals (95 % CIs) were estimated according to Breslow and Day (Breslow and Day, 1980). A subgroup analysis was performed excluding those patients with negative culture results. Multivariable logistic regression models for treatment failure were fitted according to the two different treatment duration schedules among patients with Gram-positive and Gram-negative bacterial infections. Given the limited number of events, Firth penalized logistic regression was used to reduce small-sample bias and potential separation issues. To address the potential risk of immortal time and selection bias related to treatment duration, we performed prespecified landmark sensitivity analyses at 8 and 12 weeks. For these analyses, we restricted the population to patients who had not experienced the outcome at each landmark time. This approach allows comparison of alternative antibiotic duration strategies among patients who were still at risk at the landmark time, thereby reducing immortal time bias arising from the time-dependent nature of treatment duration.

We performed statistical analyses using Stata 16.1 (Stata Corp., College Station, TX, USA). All statistical tests were two-sided, and an alpha error of 0.05 was accepted.

## 3 Results

In the study period 180 patients were diagnosed with a DSII. After applying the inclusion and exclusion criteria, 161 patients were analysed (Fig. 1). The diagnostic criteria used to define infection in the study cohort are detailed in Table S1 in the Supplement.

**Figure 1:**
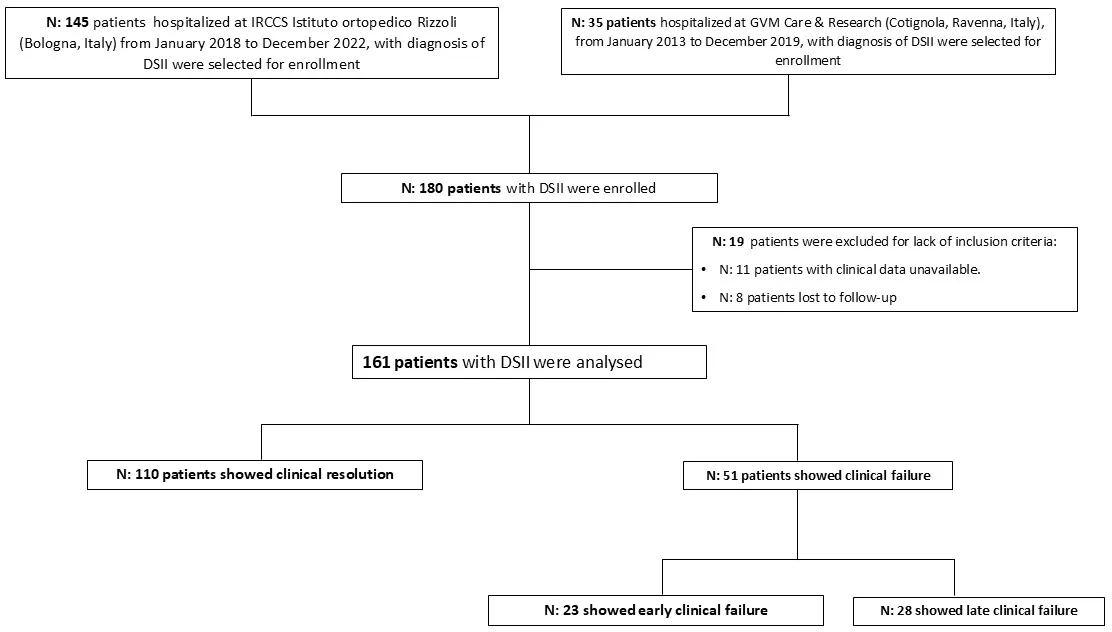
Study flow chart.

Overall, patients showed a median age of 62 years (IQR: 51–69), 87 (55.1 %) were male, and the median CCI was 3 (IQR: 1–4) (Table 1).

Spinal instrumental surgery was performed due to degenerative disease in 47 patients (29.2 %), scoliosis in 47 (29.2 %), and cancer in 31 (19.3 %). DSIIs were diagnosed at a median of 27 d (IQR: 13–41) after primary surgery and occurred more frequently in patients undergoing extensive spinal instrumentation from the cervical to the sacral region. Specifically, patients were diagnosed with early (by the first 3 months), delayed (from 3 to 24 months), and late (more than 24 months) DSII in 70.2 %, 16.5 %, and 13.3 % of cases, respectively. Wound dehiscence was the main clinical presentation (95, 59 %). Polymicrobial infection was diagnosed in 42 (26.4 %) patients. Gram-positive microorganisms accounted for the majority of DSII cases (91, 60.3%). Among Gram-negative rods, *Pseudomonas aeruginosa* (16/51, 31.4 %) and *Escherichia coli* (14/51, 27.5 %) were the most frequent pathogens isolated. As regards antibiotic treatment, the median treatment duration was 82 d (IQR: 43–96). Antibiotics used for empirical and targeted treatment are shown in Fig. 2.

**Figure 2:**
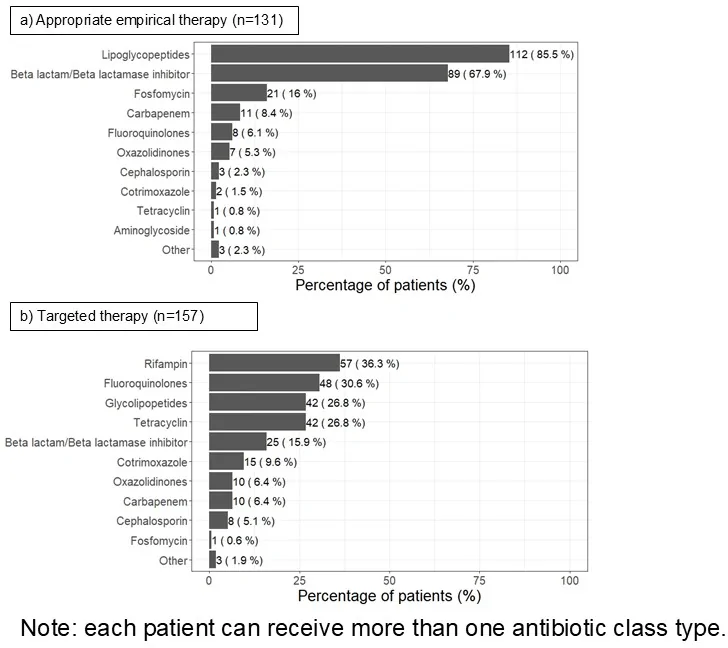
Distribution of antibiotic class of appropriate empirical and targeted therapy. Note: each patient can receive more than one antibiotic class type.

Overall, rifampin was used in 57/161 (35.4 %) of targeted treatment regimens. Similarly, quinolones were used for DSII due to Gram-negative bacteria in 6/51 (11.8 %) and 28/51 (54.9 %) of empirical and targeted regimens, respectively. Inappropriate empirical therapy was administered in 19/150 (13 %) patients.

All enrolled patients underwent surgical treatment. Specifically, removal of the spinal implant without reimplantation, one-stage debridement with implant substitution, and surgical debridement without implant replacement were performed in 11/161 (6.8 %), 57/161 (35.4 %), and 93/161 (57.8 %) patients, respectively. Repeat surgical debridement was required in 31 (19 %) patients. The median time from DSII diagnosis to surgical debridement was 6.5 d (IQR: 2–17.5).

Overall, there were 51 (31.7 %) patients with treatment failure. Among them, we observed the reopening of the wound in 33 patients (64.7 %), onset of pain/clinical impairment in 4 (7.8 %), a new onset of a fistula in 11 (21.5 %). Finally, 3 (5.9 %) patients died due to DSII.

The comparison of patients with and without treatment failure is reported in Table 1. Patients with treatment failure showed higher age (66 (IQR: 59–71) versus 60 (IQR: 49–69), p=0.004 ), more use of corticosteroid treatment (5 (9.8 %) versus 2 (1.8 %), p=0.033 ), and cancer as the main condition brought to spinal instrumentation (15 (29.4 %) versus 16 (14.6 %), p=0.026 ). Conversely, appropriate empirical antimicrobial therapy was more frequently recorded in patients with clinical cure (95 (92.2 %) versus 36 (76.6 %), p=0.008 ).

Although no statistically significant differences were observed in failure rates according to the type of surgical treatment or the timing of its performance relative to infection diagnosis, we noted that patients with early failure (i.e. treatment failure diagnosed during antimicrobial therapy) more frequently underwent surgical debridement without removal or replacement of the spinal instrumentation (i.e. a conservative approach) (Table S2). Furthermore, DSII due to Gram-negative aetiology was observed more frequently in patients with treatment failure compared to patients with clinical cure (25 (52.1 %) versus 26 (25.2 %), p=0.004 ). The unadjusted distribution of rifampin and quinolone exposure according to treatment outcome is reported in Table S3.

Multivariable analyses were carried out to evaluate risk factors for treatment failure in two separate analyses, using treatment duration cutoffs of 8 weeks and 12 weeks, respectively. Patients with early failure were excluded from multivariable analyses, and only those who completed the full antibiotic course were analysed to assess the impact of antibiotic treatment on clinical cure while excluding the influence of potentially ineffective surgical source control. This analysis did not reveal a significant impact of the treatment schedule on clinical outcomes (Table 2). Landmark sensitivity analyses at 8 and 12 weeks were performed to assess the robustness of the association between treatment duration and outcome while minimizing immortal time bias. The results were overall consistent with the primary analyses, with no significant association between prolonged therapy duration and improved outcomes at either landmark. Effect estimates for the other covariates were generally similar in direction and magnitude, although confidence intervals remained wide due to the limited number of events (Tables S4 and S5). At the 8-week landmark, 90 patients were included, of which 22 experienced treatment failure. At the 12-week landmark, 75 patients were included, of which 20 experienced treatment failure. Excluding those with a negative culture result at the 8-week landmark, 83 patients were included, with 21 treatment failures. At the 12-week landmark, 70 patients were included, with 19 treatment failures.

Gram-negative etiology was confirmed as an independent risk factor for treatment failure in both schedules (8 weeks of treatment: OR 4.20, 95 % CI 1.08–16.38, p=0.039 ; 12 weeks of treatment: OR 4.46, 95 % CI 1.15–17.23, p=0.030 ). Conversely, the administration of quinolones was associated with a lower risk of treatment failure (8 weeks: OR 0.11, 95 % CI 0.02–0.71, p=0.020 ; 12 weeks: OR 0.11, 95 % CI 0.02–0.64, p=0.015 ). Similar findings were observed in subgroup analyses conducted among patients with Gram-positive and Gram-negative bacterial infections, excluding DSII cases with negative cultures (Table 3).

## 4 Discussion

We analysed a large cohort of patients with DSII to define risk factors for treatment failure with duration of antimicrobial therapy as the main exposure. Patients who experienced treatment failure more frequently had cancer as the primary indication for surgery and a Gram-negative etiology of DSII. The role of Gram-negative pathogens as a risk factor for treatment failure was confirmed in the multivariable analysis. No significant differences were observed between the different treatment duration schedules. In contrast, the use of quinolones was associated with better outcomes.

**Table 1:** Comparison of patients with and without treatment failure of DSII.

	Total	Clinical	Treatment	p value
	N=161	cure	failure	
		N=110	N=51	
		(68.3 %)	(31.7 %)	
Demographics				
Age (years), median (IQR)	62 (51–69)	60 (49–69)	66 (59–71)	0.004
Gender (male, n=158 )	87 (55.1)	57 (53.3)	30 (58.8)	0.512
BMI, median (IQR, n=144 )	26 (23–31)	26 (23–28)	26 (23–31)	0.504
Underling disease				
Myocardial infarction	5 (3.1)	1 (0.9)	4 (7.8)	0.035
Congestive heart failure	4 (2.5)	3 (2.7)	1 (2.0)	0.999
Peripheral vascular disease	16 (9.9)	12 (10.9)	4 (7.8)	0.778
CVA/TIA	10 (6.2)	7 (6.4)	3 (5.9)	0.999
Dementia	2 (1.2)	2 (1.8)	0 (0.0)	0.999
Chronic obstructive pulmonary diseases (COPD)	12 (7.5)	5 (4.6)	7 (13.7)	0.053
Diabetes with organ damage	4 (2.5)	3 (2.7)	1 (2.0)	0.999
End-stage liver disease	1 (0.6)	1 (0.9)	0 (0.0)	0.999
Leukaemia	2 (1.2)	1 (0.9)	1 (2.0)	0.535
Lymphoma	3 (1.9)	2 (1.8)	1 (2.0)	0.999
Moderate to severe CKD	4 (2.5)	3 (2.7)	1 (2.0)	0.999
Localized solid cancer	16 (9.9)	8 (7.3)	8 (15.7)	0.154
Metastatic solid cancer	17 (10.6)	8 (7.3)	9 (17.7)	0.046
Charlson comorbidities index (CCI), median (IQR)	3 (1–4)	3 (1–4)	3 (2–6)	0.002
Immunosuppression				
Hematopoietic stem cell transplantation	2 (1.2)	1 (0.9)	1 (2.0)	0.535
Solid organ transplantation	1 (0.6)	1 (0.9)	0 (0.0)	0.999
Corticosteroids	7 (4.4)	2 (1.8)	5 (9.8)	0.033
Other	7 (4.4)	6 (5.5)	1 (2.0)	0.433
Reason of surgery				
Degenerative pathology	47 (29.2)	33 (30.0)	14 (27.5)	0.741
Cancer	31 (19.3)	16 (14.6)	15 (29.4)	0.026
Spondylodiscitis	9 (5.6)	7 (6.4)	2 (3.9)	0.720
Scoliosis	47 (29.2)	33 (30.0)	14 (27.5)	0.741
Other	22 (13.7)	16 (14.6)	6 (11.8)	0.633
Anatomical region of spinal instrumentation ( n=125 )				0.009
Cervical, thoracic, lumbar, and sacral	3 (2.4)	0 (0.0)	3 (7.3)	
Cervical, thoracic	4 (3.2)	4 (4.8)	0 (0.0)	
Thoracic, lumbar, and sacral	36 (28.8)	18 (21.4)	18 (43.9)	
Thoracic, lumbar	21 (16.8)	17 (20.2)	4 (9.8)	
Thoracic	15 (12.0)	9 (10.7)	6 (14.6)	
Lumbar, sacral	26 (20.8)	20 (23.8)	6 (14.6)	
Lumbar	20 (16.0)	16 (19.0)	4 (9.8)	
Clinical presentation				
Wound dehiscence	95 (59.0)	63 (57.3)	32 (62.8)	0.511
Fistula	31 (19.3)	22 (20.0)	9 (17.7)	0.725
Pain/clinical impairment	31 (19.3)	21 (19.0)	10 (19.6)	0.938
Fever	61 (37.9)	40 (36.4)	21 (41.2)	0.558
Other	24 (14.9)	18 (16.4)	6 (11.8)	0.446
Characteristics of infection				
Polymicrobial infection	42 (26.4)	25 (23.2)	17 (33.3)	0.174

**Table 1:** Continued.

	Total	Clinical	Treatment	p value
	N=161	cure	failure	
		N=110	N=51	
		(68.3 %)	(31.7 %)	
Etiological agent group ( n=151 )				0.004
Gram-positive	91 (60.3)	69 (67.0)	22 (45.8)	
*Coagulase negative staphylococcus*	41 (45.0)	32 (46.4)	9 (40.9)	
*Staphylococcus aureus*	35 (38.5)	23 (33.3)	12 (54.6)	
*Enterococcus faecalis*	5 (5.5)	5 (7.2)	0 (0.0)	
*Cutibacterium acnes*	8 (8.8)	7 (10.1)	1 (4.5)	
*Corynebacterium* spp.	1 (1.1)	1 (1.5)	0 (0.0)	
*Streptococcus* spp.	1 (1.1)	1 (1.5)	0 (0.0)	
Gram-negative	51 (33.8)	26 (25.2)	25 (52.1)	
*Pseudomonas* spp.	16 (31.4)	11 (42.3)	5 (20.0)	
*Escherichia coli*	14 (27.5)	7 (26.9)	7 (28.0)	
*Proteus* spp.	6 (11.8)	3 (11.5)	3 (12.0)	
*Klebsiella pneumoniae*	5 (9.8)	2 (7.7)	3 (12.0)	
*Enterobacter* spp.	3 (5.9)	1 (3.8)	2 (8.0)	
*Morganella* spp.	3 (5.9)	1 (3.8)	2 (8.0)	
*Serratia* spp.	2 (3.9)	0 (0.0)	2 (8.0)	
*Acinetobacter* spp.	1 (1.9)	0 (0.0)	1 (4.0)	
*Other Gram-negative*	1 (1.9)	1 (3.8)	0 (0.0)	
Negative culture	9 (6.0)	8 (7.8)	1 (2.1)	
Surgical procedure				
Removal of spine implant without repositioning	11 (6.8)	6 (5.5)	5 (9.8)	0.198
One-stage debridement of spine implant (removed and repositioned in a single intervention)	57 (35.4)	40 (36.4)	17 (33.3)	0.708
Surgical debridement without replacement of spine implant	93 (57.8)	64 (58.2)	29 (56.9)	0.875
Time from infection diagnosis to surgical procedure (days), median (IQR)	6.5 (2–17.5)	6 (2–19)	7.5 (2.7–17.5)	0.603
Antibiotic compliance				
Drug discontinued due to intolerance ( n=147 )	40 (27.2)	27 (27.6)	13 (26.5)	0.896
Adverse reaction ( n=148 )	25 (16.9)	17 (17.2)	8 (16.3)	0.897
Antibiotic therapy				
Appropriate empirical therapy ( n=150 )	131 (87.3)	95 (92.2)	36 (76.6)	0.008
Appropriate therapy duration (days), median (IQR, n=150 )	82 (43–96)	83 (57–97)	61 (30–89)	0.011

All values given are n (%) unless otherwise stated. Data are available for 161 patients unless otherwise stated. Abbreviations: BMI, body mass index; CVA, cerebral vascular accident; TIA, transient ischemic attack; CKD, chronic kidney disease; HIV, human immunodeficiency virus.

**Table 2:** Firth multivariable logistic regressions for treatment failure according to 8 and 12 weeks of antibiotic treatment duration ( n=127 ).

	8-week cutoff^a^ treatment			12-week cutoff^b^ treatment	
	duration			duration	
	OR (95 % CI)	p value		OR (95 % CI)	p value
Appropriate therapy duration (weeks)					
≤8 (or ≤12 )	Reference			Reference	
>8 (or >12 )	0.85 (0.29–2.50)	0.762		0.99 (0.37–2.65)	0.988
Cancer as reason of surgery					
No	Reference			Reference	
Yes	3.00 (0.92–9.78)	0.069		2.96 (0.91–9.55)	0.070
Age (years)					
≤63	Reference			Reference	
>63	1.70 (0.60-4.77)	0.315		1.65 (0.60–4.53)	0.334
Charlson comorbidities index (CCI)					
≤3	Reference			Reference	
> 3	2.78 (0.97–7.94)	0.056		2.81 (0.99–7.99)	0.053
Polymicrobial infection					
No	Reference			Reference	
Yes	1.00 (0.30–3.29)	0.999		0.99 (0.30–3.26)	0.984
Targeted therapy					
Neither quinolones nor rifampin	Reference			Reference	
Quinolones	0.11 (0.02–0.71)	0.020		0.11 (0.02–0.64)	0.015
Rifampin	1.07 (0.32–3.57)	0.915		1.05 (0.32–3.47)	0.936
Quinolones and rifampin	0.27 (0.05–1.42)	0.122		0.25 (0.05–1.33)	0.105
Etiological agent group					
Gram-positive	Reference			Reference	
Gram-negative	4.20 (1.08–16.38)	0.039		4.46 (1.15–17.23)	0.030
Negative culture	1.40 (0.20–9.94)	0.734		1.38 (0.19–9.83)	0.748

^a^ A total of 35 patients were treated for ≤8 weeks and 92 for >8 weeks. ^b^ A total of 73 patients were treated for ≤12 weeks and 54 for >12 weeks. Note: patients with early clinical failure ( n=22 , i.e. clinical failure diagnosed during antibiotic treatment), a patient with early failure and missing etiological information ( n=1 ), patients without information on etiological agent group ( n=8 ), and patients with missing treatment start/end dates and/or outcome dates ( n=3 ) were excluded from the main analysis.

**Table 3:** Firth multivariable logistic regressions for treatment failure according to antibiotic treatment duration among patients with Gram-positive and Gram-negative bacterial infections ( n=118 ).

	8-week cutoff^a^ treatment			12-week cutoff^b^ treatment	
	duration			duration	
	OR (95 % CI)	p value		OR (95 % CI)	p value
Appropriate therapy duration (weeks)					
≤8 (or ≤12 )	Reference			Reference	
>8 (or >12 )	0.81 (0.27–2.42)	0.710		0.92 (0.34–2.51)	0.877
Cancer as reason of surgery					
No	Reference			Reference	
Yes	3.12 (0.96–10.16)	0.058		3.06 (0.95–9.85)	0.060
Age (years)					
≤63	Reference			Reference	
>63	1.61 (0.57–4.53)	0.371		1.55 (0.56–4.28)	0.400
Charlson comorbidities index (CCI)					
≤3	Reference			Reference	
>3	2.37 (0.82–6.89)	0.112		2.41 (0.83–6.98)	0.105
Polymicrobial infection					
No	Reference			Reference	
Yes	1.04 (0.32–3.37)	0.950		1.03 (0.32–3.37)	0.958
Targeted therapy					
Neither quinolones nor rifampin	Reference			Reference	
Quinolones	0.12 (0.12–0.79)	0.027		0.12 (0.02–0.72)	0.021
Rifampin	1.23 (0.36–4.22)	0.745		1.20 (0.36–4.04)	0.769
Quinolones and rifampin	0.29 (0.06–1.54)	0.147		0.28 (0.03–1.45)	0.128
Etiological agent group					
Gram-positive	Reference			Reference	
Gram-negative	4.96 (1.08–16.07)	0.039		4.37 (1.14–16.78)	0.032

^a^ A total of 34 patients were treated for ≤8 weeks and 84 for >8 weeks. ^b^ A total of 69 patients were treated for ≤12 weeks and 49 for >12 weeks. Note: patients with early treatment failure ( n=22 , i.e. failure diagnosed during antibiotic treatment), a patient with early failure and missing etiological information ( n=1 ), patients with missing treatment start/end dates and/or outcome dates ( n=3 ), negative culture results ( n=9 ), and patients without information on etiological agent group ( n=8 ) were excluded from this subgroup analysis.

Our cohort's epidemiology aligns with the existing literature, where degenerative spinal disease and scoliosis are leading causes for spinal instrumentation (Buser et al., 2018). However, a significant proportion of our patients (19.3 %) underwent spinal instrumentation for cancer and experienced treatment failure, likely reflecting the impaired ability of cancer patients to control infection due to immunosuppression, chemotherapy, disruption of physical barriers, and malnutrition (Seo et al., 2021).

Gram-positive organisms were the most frequent isolated pathogens in our cohort, as expected. However, Gram-negative bacteria, though less frequent, accounted for 34 % of infections, a proportion consistent with prior studies (de la Hera et al., 2021; Margaryan et al., 2020) and associated with a higher risk of treatment failure (Cunningham et al., 2017; Margaryan et al., 2020; Tsilika et al., 2025). Current understanding of implant-associated spinal infections and bone and joint infections caused by Gram-negative bacteria remains limited (Galanakis et al., 1997; Graham et al., 2013; Koutserimpas et al., 2018). However, it is well established that patients with malignancies are predisposed to Gram-negative infections due to immune dysfunction, chemotherapy-induced mucositis, the use of invasive devices, and prolonged hospital stays (Amanati et al., 2021; Islas-Muñoz et al., 2018).

Another important factor in evaluating DSII outcomes is the role of antibiotics with activity against biofilm. Quinolones and rifampin are among the most used for bone and joint infections (Lora-Tamayo et al., 2019; Pupaibool, 2024). In our study, quinolones seem to be associated with a reduced risk of treatment failure, in line with previous reports, particularly in cases with a Gram-negative etiology (Martínez-Pastor et al., 2009; Rodríguez-Pardo et al., 2014). However, the association between quinolone use and improved outcomes should be interpreted cautiously. Although Gram-negative infections seem associated with worse outcomes, quinolones were likely used in selected patients with susceptible pathogens and optimized targeted therapy. Thus, the observed association may reflect appropriate antimicrobial selection within a higher-risk subgroup rather than a true causal effect. Moreover, differences in surgical source control may have influenced both treatment choice and outcomes. Despite excluding early failures, residual confounding cannot be ruled out. Conversely, we did not demonstrate a protective effect of rifampin. However, evidence for rifampin's efficacy in bone and joint infections is limited. While retrospective studies have suggested benefits in prosthetic joint infections (Kruse et al., 2022), randomized controlled trials have failed to confirm a clear advantage (Pushkin et al., 2016; Zimmerli et al., 1998).

Currently, there are no clear guidelines on the optimal antibiotic treatment duration for DSII. From previous experiences, it ranges between 6–24 weeks (Benavent et al., 2021; Fernandez-Gerlinger et al., 2019; de la Hera et al., 2021; Karamian et al., 2022; Spatenkova et al., 2021; Tai et al., 2024). In our cohort, the median duration of antibiotic treatment was 11.7 weeks (IQR: 6–13). To investigate the efficacy of antibiotic therapy duration and minimize the impact of the surgical procedure on the outcome, we excluded early treatment failures (i.e. failure during antibiotic treatment). We investigated different antibiotic therapy schedules without finding a significant association between treatment duration and outcome in our exploratory analyses; however, these findings should not be interpreted as a direct comparison between different treatment durations. More studies are needed to determine the optimal antibiotic treatment duration for Gram-negative spinal infections.

Our study has several limitations. First, it involves two centres in the same geographic area, thus limiting the generalizability of the results. However, the standardized approach to patient care adopted in both centres, particularly regarding ID management, may enhance the reliability of our results. Notably, treatment duration was managed by the physician's personal assessment and not by study procedures. Consequently, confounding by indication cannot be excluded, as patients with more severe infections or poorer clinical conditions may have been preferentially treated with longer antibiotic regimens. A further limitation of our study is the rate of negative culture infections, higher than what has been reported in the literature (de la Hera et al., 2021). To mitigate this limitation, we conducted a sub-analysis of risk factors for treatment failure only in the population with a confirmed microbial etiology. Another limitation of this study is the difficulty in disentangling the relative impact of medical treatment and surgical intervention on clinical outcomes. Surgical factors, including type of procedure, number of debridements, and complexity of the surgery, are major determinants of outcome in DSII. However, these variables were not included in the multivariable models due to incomplete data and concerns of overfitting, and this may have resulted in residual confounding. We attempted to mitigate this issue by excluding patients with early failure from the analysis. However, this approach may have introduced both selection bias and immortal time bias. To mitigate the potential immortal time bias inherent to analyses of treatment duration, we additionally performed landmark sensitivity analyses at 8 and 12 weeks. These analyses yielded findings consistent with the primary models, supporting the robustness of the overall conclusions, although residual confounding and selection bias cannot be fully excluded in this observational setting. However, given the descriptive aim of the study, we focused on exploratory associations using logistic regression models rather than applying more complex time-to-event analyses. In addition, detailed information on the surgical procedure (including surgical magnitude, number of instrumented levels, presence of interbody devices) was not consistently available, potentially limiting adjustment for factors influencing infection severity and treatment complexity. In addition, the relatively low number of events compared to the number of variables included in the multivariable models may have increased the risk of overfitting and produced unstable estimates with wide confidence intervals. Therefore, these findings should be interpreted with caution and considered exploratory.

Another limitation of this study is the relatively short follow-up period for a DSII. However, this duration was chosen to ensure the study's feasibility and is consistent with the follow-up periods used in previous studies (Benavent et al., 2021; Fernandez-Gerlinger et al., 2019). Finally, being a retrospective study, there are missing data and challenges in precisely defining outcomes that are not clearly measurable from the retrospective documentation. However, a careful data review was attempted to minimize this issue.

## 5 Conclusion

Our study shows that patients with cancer as the main indication for spinal instrumentation and DSII due to Gram-negative rods seem to have a worse outcome more frequently. Our findings suggest that while treatment duration remains an important consideration, clinical outcome is more strongly influenced by the type of microorganism and the use of appropriate antibiotics rather than the treatment schedule. However, further studies, preferably prospective, are needed to confirm these findings.

## Supplement

10.5194/jbji-11-601-2026-supplement The supplement related to this article is available online at https://doi.org/10.5194/jbji-11-601-2026-supplement.

## Data Availability

The data are not publicly available due to the data-sharing policies of the participating universities and research institutions. De-identified data may be obtained from the corresponding author (Renato Pascale: renato.pascale2@unibo.it) upon reasonable request and subject to institutional approval.
