# Supplementary material for: Risk factors for treatment failure of surgical site deep infections following spinal instrumentation: multicentre observational study (DeFEndO study)
Source: J Bone Jt Infect. 2026 Oct 6;11(5):601–11. doi: 10.5194/jbji-11-601-2026 (PMC13640963; doi:10.5194/jbji-11-601-2026)
Supplement: Supplementary file 1 [file jbji-11-601-2026-supplement.pdf]

Supplement of J. Bone Joint Infect., 11, 601–611, 2026
https://doi.org/10.5194/jbji-11-601-2026-supplement
© Author(s) 2026. CC BY 4.0 License.

JBJI
Journal of Bone and Joint Infection
Open Access

Supplement of

Risk factors for treatment failure of surgical site deep infections following spinal instrumentation: multicentre observational study (DeFEndO study)

Renato Pascale et al.

Correspondence to: Renato Pascale (renato.pascale2@unibo.it) and Sara Tedeschi (sara.tedeschi5@unibo.it)

The copyright of individual parts of the supplement might differ from the article licence.

Supplement

Sect. S1 Variables and Definitions

DSII diagnosis was established according to a recent Delphi-based consensus definition endorsed by the Musculoskeletal Infection Society (MSIS) and the European Bone and Joint Infection Society (EBJIS) (Shaw et al., 2025). Specifically, DSII was defined using a hierarchical combination of microbiological, clinical, radiological, laboratory, intraoperative, and histological criteria. A stand-alone microbiological criterion, defined as the isolation of the same microorganism from at least two deep operative site specimens (tissue, fluid, or implant-associated samples), was considered sufficient to establish a definite infection. In the absence of this criterion, diagnosis relied on supporting findings categorized as primary or secondary. Primary supporting criteria included inflammatory wound features (e.g., erythema, purulence, dehiscence, or sinus tract), identification of a pathogen from a single deep operative specimen or positive blood cultures without an alternative source, intraoperative evidence of purulence or abscess, and imaging findings consistent with deep infection (e.g., epidural abscess, discitis, or vertebral osteomyelitis). Secondary supporting criteria comprised less specific findings such as deep fluid collections or hardware loosening on imaging, elevated inflammatory biomarkers (CRP and/or ESR) without another cause, intraoperative evidence of hardware failure or pseudarthrosis, and histological evidence of acute or chronic inflammation. Definite infection was defined by the presence of the stand-alone criterion or at least three primary supporting criteria, whereas probable infection was defined by either two primary criteria or one primary plus at least two secondary criteria.

In our surgical approach, three to six specimens for microbiological culture were sampled from different sites for all patients at time of surgical procedures (Tai et al., 2024). Furthermore, histological samples were collected for all patients undergoing surgical debridment. DSII diagnosis was confirmed in case of at least five neutrophils per high-power field (Zimmerli and Ochsner, 2003). The choice of surgical strategy (implant retention, removal, or substitution) was primarily based on the assessment of spinal stability. Patients with preserved spinal stability were preferentially managed with complete implant removal, whereas those with spinal instability underwent implant substitution to maintain structural support. Additionally, a more conservative approach consisting of surgical debridement without implant removal was generally adopted in cases of early infection following spinal implantation or when patient frailty warranted a minimally invasive strategy. Staged revision procedures were not routinely performed in our cohort. Local intraoperative antibiotic measures (i.e. intrawound antibiotics, local antibiotic carriers, or antibiotic irrigation strategies) were not used as part of the surgical management.

Appropriate empirical antibiotic therapy was defined as a therapy administered before the susceptibility report was available and containing at least one in vitro active drug (according to the susceptibility pattern of the isolate). Antibiotic treatment administered according to susceptibility results was considered as targeted antibiotic therapy. Duration of antibiotic treatment was defined as the number of consecutive days during which the patient received an appropriate antibiotic regimen.

Clinical cure was defined as resolution of clinical symptoms of DSII after surgical treatment and a complete course of appropriate antibiotic therapy, confirmed at the end of the follow-up period.

Clinical impairment was defined as a significant deviation, loss, or loss of use of in an individual with a health condition, disorder, or disease (Brigham, 2019). Specifically for patients with DSII, clinical impairment encompasses the loss of structural integrity of the spine and functional deterioration, including chronic pain, impaired mobility, and potential neurological deficits.

Variables collected for all patients included age, sex, Charlson comorbidity index (CCI) (Charlson et al., 1987), immunosuppression condition defined as haematopoietic stem cell transplantation, solid organ transplantation, chronic corticosteroids use. The reason for spinal instrumental implantation was collected and defined as degenerative disease, cancer, spondilodyscitis, scoliosis and other causes not classifiable with the previous ones. Gram-negative infection was defined in presence of culture of intraoperative tissue sample and/or sonication fluid yielding Gram-negative rods.

REFERENCES

Brigham, C.R., 2019. Common Metrics for Impairment Ratings. Phys. Med. Rehabil. Clin. N. Am. 30, 533–540. https://doi.org/10.1016/j.pmr.2019.03.003

Charlson, M.E., Pompei, P., Ales, K.L., MacKenzie, C.R., 1987. A new method of classifying prognostic comorbidity in longitudinal studies: development and validation. J. Chronic Dis. 40, 373–383. https://doi.org/10.1016/0021-9681(87)90171-8

Shaw, J.D., Nelson, S.B., Simpfendorfer, C., Berbari, E.F., Henry, M.W., Kleck, C.J., Savage, J., Agarwal, N., Oyekan, A.A., Rodrigues-Pinto, R., Wouthuyzen-Bakker, M., Tande, A.J., 2025. New Definition for Postoperative Spine Infection (PSI): From the Workgroup of the Musculoskeletal Infection Society (MSIS) and the European Bone & Joint Infection Society (EBJIS). Spine. https://doi.org/10.1097/BRS.0000000000005604

Tai, D.B.G., Patel, R., Lovecchio, F., Kwee, T., Wouthuyzen-Bakker, M., 2024. State-of-the-Art Review: Diagnosis and Management of Spinal Implant Infections. Clin. Infect. Dis. Off. Publ. Infect. Dis. Soc. Am. 79, e65–e71. https://doi.org/10.1093/cid/ciae436

Zimmerli, W., Ochsner, P.E., 2003. Management of infection associated with prosthetic joints. Infection 31, 99–108. https://doi.org/10.1007/s15010-002-3079-9

Table S1: Diagnostic criteria to define DSSI infection in the study cohort (Shaw et al., 2025)

**Criteria**	**Category**		**Number: 161 (%)**
Stand-alone	Microbiology	Identification of a phenotypically indistinguishable organism from TWO or more deep operative site specimens (deep tissue, fluid or implant/sonicate) by culture (or demonstration of an identical pathogen in culture and in a validated molecular diagnostic assay)	125 (77.6)
Primary Supporting	Wound Features	Inflammatory wound including sinus tract,	126 (78.2)

		erythema, purulence, prolonged drainage or dehiscence	
	Microbiology	Identification of pathogen from ONE deep operative site specimen. (Positive culture or molecular diagnostic from deep tissue, fluid or implant / sonicate)<br><br>Positive blood cultures without alternative source	17 (10.5)
	Intraoperative findings	Purulence or Abscess	131 (81.3)
	Imaging	Epidural abscess, discitis or vertebral osteomyelitis	120 (74.5)
Secondary Supporting	Imaging	Deep fluid collection or hardware loosening	20 (12.4)
	Laboratory	Elevated CRP and/or ESR without alternative cause	79 (49.1)
	Intraoperative findings	Pseudarthrosis, loose or broken hardware or metal staining	130 (80.7)
	Histology	Acute / chronic inflammation	150 (93.2)

Table S2. Characteristics of patients with early and late treatment failure.

	**Early treatment failure N=23**	**Late treatment failure N=28**
**Clinical failure**		
Reopening of the wound	17 (77.2%)	16 (57.1%)
Onset of the fistula	4 (17.4%)	7 (25%)
Onset of Pain/ Clinical impairment	1 (4.5%)	3 (11.5%)
Death	1 (4.5%)	2 (7.7%)
**Surgical Procedure**		
Removal of spine implant without repositioning	2 (8.6%)	3 (11%)
One-stage debridement of spine implant (removed and repositioned in a single intervention)	7 (31.8%)	10 (35.7%)
Surgical debridement without replacement of spine implant	14 (60.8%)	15 (57.7%)

Note: early treatment failure refers to treatment failure diagnosed during antibiotic treatment; late treatment failure refers to clinical failure recorded after the conclusion of antibiotic treatment

Table S3. Unadjusted distribution of rifampin and quinolones according to treatment outcome. **Antibiotic**

Total	N=161 (%)	Clinical Cure N=110 (%)	Treatment Failure N=51 (%)	p-value
Targeted therapy				0.501
Neither Quinolones nor Rifampin	76 (47.2)	49 (44.6)	27 (52.9)	
Quinolones	28 (17.4)	18 (16.4)	10 (119.6)	
Rifampin	37 (23.0)	27 (24.6)	10 (19.6)	
Quinolones and Rifampin	20 (12.4)	16 (14.6)	4 (7.8)	

Table S4. Multivariable penalized logistic regression (Firth correction) for treatment failure at 8 and 12 weeks, based on landmark analyses at 8 and 12 weeks.

	8-week cutoff[a] treatment duration		12-week cutoff[b] treatment duration	
	OR (95% CI)	p-value	OR (95% CI)	p-value
Appropriate therapy duration (weeks)				
≤8 (or ≤12)	Reference		Reference	
>8 (or >12)	0.58 (0.13-2.63)	0.479	0.87 (0.26-2.85)	0.812
Cancer as reason of surgery				
No	Reference		Reference	
Yes	2.23 (0.63-7.81)	0.211	2.13 (0.55-8.31)	0.276
Age (years)				
≤63	Reference		Reference	
>63	2.45 (0.80-7.51)	0.016	1.99 (0.62-6.44)	0.250
Charlson Comorbidities Index (CCI)				
≤3	Reference		Reference	
>3	2.33 (0.74-7.33)	0.148	2.80 (0.81-9.67)	0.104
Polymicrobial infection				
No	Reference		Reference	
Yes	1.25 (0.25-6.26)	0.783	1.28 (0.24-6.75)	0.775
Targeted therapy				
Neither Quinolones nor Rifampin	Reference		Reference	
Quinolones	0.18 (0.03-1.14)	0.070	0.08 (0.01-0.77)	0.029

Rifampin	0.69 (0.19-2.47)	0.567	0.89 (0.23-3.46)	0.869
Quinolones and Rifampin	0.35 (0.07-1.63)	0.179	0.38 (0.07-2.03)	0.257
Etiological agent group				
Gram-positive	Reference		Reference	
Gram-negative	1.35 (0.23-7.74)	0.738	1.97 (0.32-11.96)	0.462
Negative Culture	0.82 (0.10-6.39)	0.847	1.07 (0.12-9.74)	0.951

[a]Patients treated for ≤8 weeks and >8 weeks are 11 and 79, respectively (n=90).

[b]Patients treated for ≤12 weeks and >12 weeks are 32 and 43, respectively (n=75).

Table S5. Multivariable penalized logistic regression (Firth correction) for treatment failure at 8 and 12 weeks, based on landmark analyses at 8 and 12 weeks among patients with Gram positive and negative bacterial infections.

	**8-week cutoff[a] treatment duration**		**12-week cutoff[b] treatment duration**	
	**OR (95% CI)**	**p-value**	**OR (95% CI)**	**p-value**
Appropriate therapy duration (weeks)				
≤8 (or ≤12)	Reference		Reference	
>8 (or >12)	0.59 (0.13-2.66)	0.495	0.84 (0.25-2.78)	0.773
Cancer as reason of surgery				
No	Reference		Reference	
Yes	2.33 (0.67-8.12)	0.183	2.26 (0.58-8.78)	0.238
Age (years)				
≤63	Reference		Reference	
>63	2.35 (0.76-7.30)	0.139	1.93 (0.59-6.36)	0.277
Charlson Comorbidities Index (CCI)				
≤3	Reference		Reference	
>3	2.00 (0.62-6.45)	0.247	2.34 (0.65-8.39)	0.191
Polymicrobial infection				
No	Reference		Reference	
Yes	1.23 (0.25-6.04)	0.793	1.28 (0.25-6.66)	0.765
Targeted therapy				
Neither Quinolones nor Rifampin	Reference		Reference	

Quinolones	0.21 (0.03-1.533)	0.096	0.09 (0.01-0.88)	0.039
Rifampin	0.75 (0.420-2.76)	0.664	1.01 (0.25-4.13)	0.986
Quinolones and Rifampin	0.38 (0.08-1.75)	0.213	0.42 (0.08-2.24)	0.309
Etiological agent group				
Gram-positive	Reference		Reference	
Gram-negative	1.36 (0.25-7.71)	0.793	2.00 (0.33-11.94)	0.449

[a]Patients treated for ≤8 weeks and >8 weeks are 11 and 72, respectively (n=83).

[b]Patients treated for ≤12 weeks and >12 weeks are 31 and 39, respectively (n=70).
